# Follow the Shoestring: A Unique Case of Bullet Extraction

**DOI:** 10.7759/cureus.14862

**Published:** 2021-05-05

**Authors:** Danier Ong, Parth M Joshi, Holly Zehfus, Timothy Havens

**Affiliations:** 1 Emergency Medicine, Saint Louis University School of Medicine, St. Louis, USA

**Keywords:** bullet wound, bullet fragment, bullet extraction, lower extremity trauma, emergency medicine and trauma

## Abstract

Firearm-related injuries and deaths remain a major issue in the United States. Gunshot injuries to the foot can be particularly difficult to manage when they occur as they can cause fractures or devastating neurovascular damage. There are limited reasons for routine bullet removal in most cases. Acute indications include wounds involving joints, palms, and soles as well as increased risk of infection, persistent pain, and lead intoxication. Here, we bring attention to a case of a gunshot wound to the left foot of a 53-year-old male, in which the bullet was able to be extracted using a shoe fiber that had become wrapped around the bullet.

## Introduction

According to the 2018 data from the Centers for Disease Control and Prevention (CDC), there were estimated to be 39,740 firearm-related deaths in the United States [1]. In addition, over 67,000 Americans are non-fatally injured by firearms each year [2]. The issue is so prevalent in American society that the CDC notes seven out of every 10 medically treated firearm injuries are the result of a firearm-related assault [1]. The effect that these injuries can have on individuals can range from hospitalization to death. Injuries to the foot are particularly challenging due to anatomical and functional considerations related to that region. A preponderance of gunshot injuries to the foot result in fracture, and there is a higher chance of intra-articular involvement with neurovascular injury [3]. Another unique consideration that must be made when a foot/ankle gunshot injury occurs is whether to retrieve the bullet. This decision is made based on the presence of motor issues, location of the lesion, and degree/exacerbation of pain [3]. The most common types of imaging used to assess gunshot injuries are x-rays and can be escalated to total body CT if there are signs of vascular damage present [4]. Here, we report a case of a gunshot wound to the left foot of a 53-year-old male, in which the bullet was able to be extracted using a shoe fiber that had become wrapped around the bullet.

## Case presentation

A 53-year-old male without known significant past medical history presented to the emergency department for a gunshot wound to the left foot. He reported leaving a bar when a shot was fired, hitting him in the foot. He noted pain and bleeding but was able to walk. His pain was worsened with palpation to the affected site. The patient denied any significant family history. His social history was significant for tobacco use of two packs per day and alcohol use of six to 12 beers a day.

**Figure 1:**
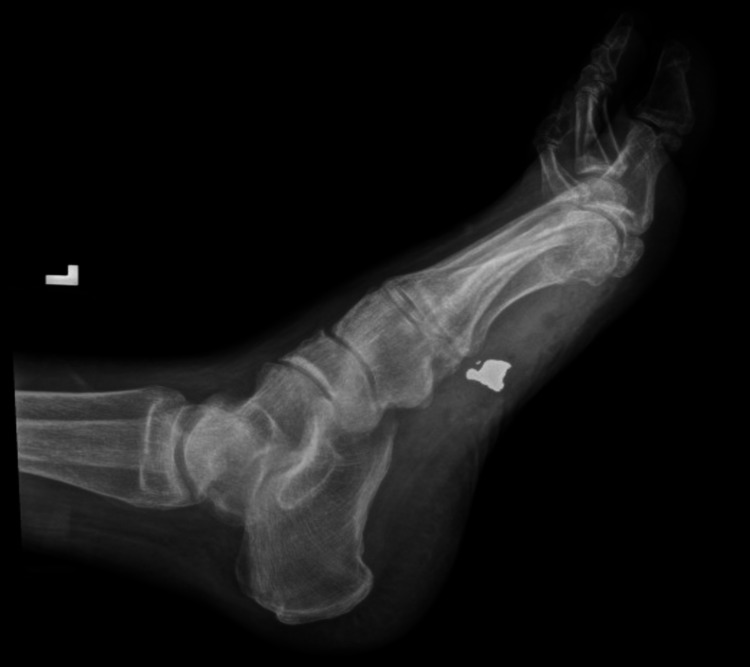
X-ray foot left lateral view (before bullet removal)

Upon arrival, blood pressure was 152/85 mmHg, pulse was 76 bpm, respiratory rate was 18 bpm, the temperature was 97.6°F, and O_2_ saturation was 100% on room air. On examination, there was a wound with associated tenderness and edema to the medial aspect of the left foot with no other findings noted. A left foot x-ray was ordered and tetanus, diphtheria, and pertussis (Tdap) vaccine were administered intramuscularly. X-ray imaging revealed a large bullet fragment in the plantar soft tissue beneath the medial cuneiform as well as punctate fragments in the medial soft tissue near the large fragment. There were no fractures noted on imaging (Figure 1).

**Figure 2:**
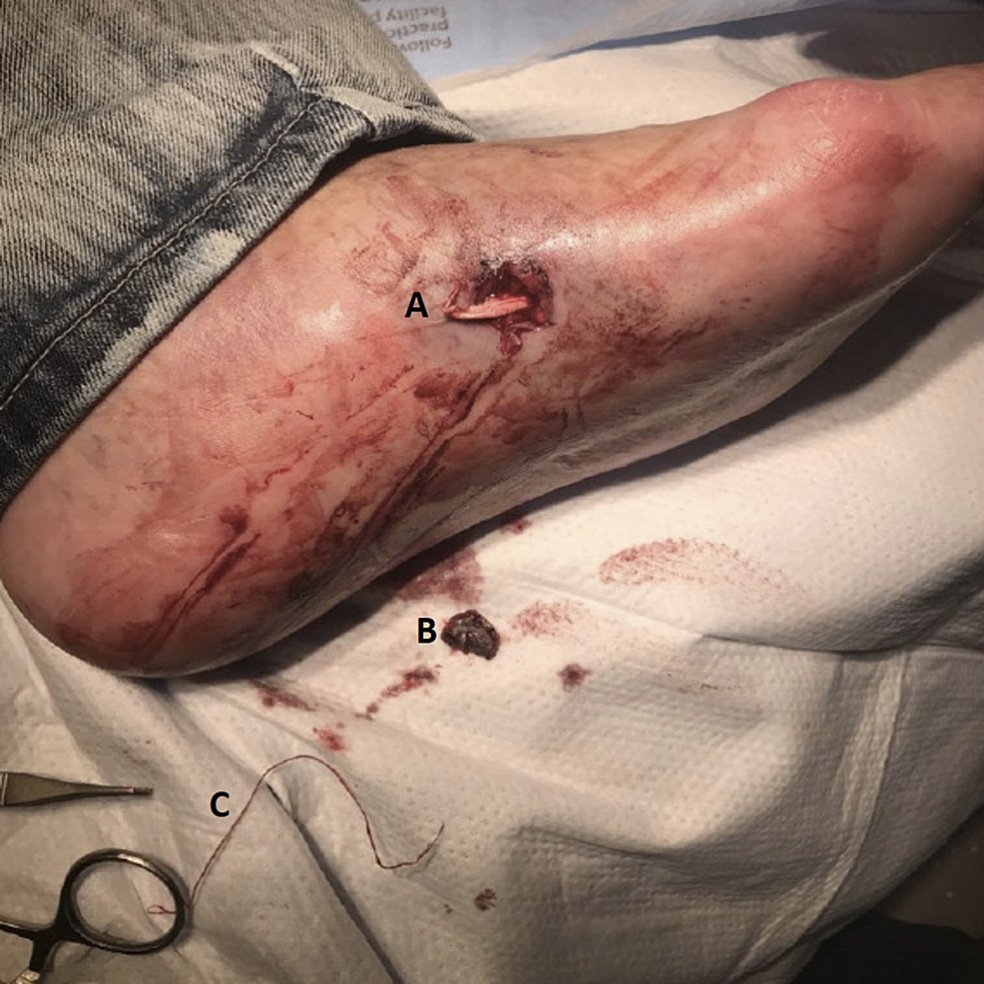
Left foot gunshot wound with a removed bullet fragment A. Gunshot wound B. Bullet fragment C. Shoe fiber that had been wrapped around bullet fragment

Lidocaine 1% with epinephrine was administered locally and removal of the bullet was attempted. The bullet path was tracked by following pieces of thread detached from the patient’s shoe, which was found to be wrapped around the bullet (Figure 2).

**Figure 3:**
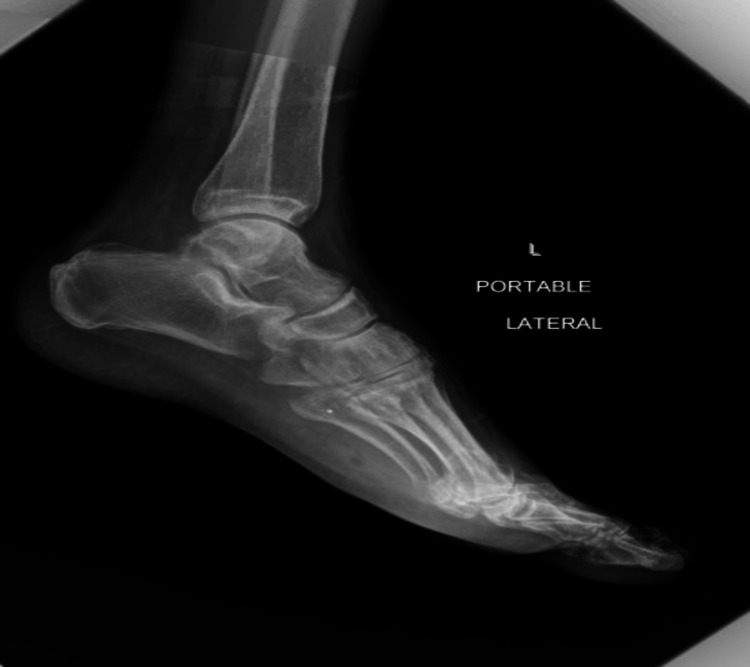
X-ray foot left lateral view (after bullet removal)

Bullet extraction was performed by pulling the shoe fibers along the bullet path. The severed tendon was visible upon extraction following bullet removal. Plantar and dorsiflexion of all toes of the left foot were intact. Orthopedic surgery was consulted and recommended a repeat x-ray which showed removal of the large bullet fragment in the plantar soft tissues (Figure 3). 

The patient was discharged with topical bacitracin and ciprofloxacin 500 mg tablet two times daily for seven days. He was provided with crutches and instruction for outpatient management with orthopedic surgery. The patient did not follow up for further management.

## Discussion

A prior systematic review of gunshot wounds and bullet removal has concluded that there is no rationale for routine bullet removal in all cases; acute indications for removal include wounds involving joints, palms, and soles as well as increased risk of infection, persistent pain, and lead intoxication [5]. Upon evaluation of our patient, it was subjectively determined that bullet extraction would be challenging. Removal of bullets within soft tissue is not typically indicated except in specific situations as previously stated. The majority of literature discussing bullet removal from the extremities involves arthroscopy for intra-articular injuries or surgical intervention involving fractures of the foot [6]. In regards to non-complicated soft tissue injures, management typically involves superficial irrigation as opposed to formal surgical irrigation and debridement [3,7,8]. The issue we faced was a non-complicated soft tissue injury, specifically affecting the foot. It has been previously noted that gunshot wounds can worsen prognosis due to the increased risk of damaging adjacent neurovascular structures [9]. Considering the proximity of the bullet to plantar vessels and nerves, this patient carried the potential for further injury from bullet migration, exacerbated via repeated movement and ambulation. Additionally, the persistent discomfort exacerbated by palpation and ambulation provided further rationale for the extraction. Based on these determinations, management recommendations were reconsidered.

The decision to manage conservatively versus operatively was facilitated by the presence of the shoe fibers. Typical bullet extraction necessitates further wound incision in a sterile, operative setting, which would have created a greater burden on the patient and his recovery. From this, it can be concluded that removal of the bullet via the shoe fibers prevented further soft-tissue damage. Furthermore, upon review of the wound site, we found evidence of tendinous injury, suggesting that opting for conservative management would have only delayed the need for future orthopedic surgical intervention.

## Conclusions

This is a unique case of bullet extraction by mechanical removal using entrapped shoe fibers surrounding a lodged bullet. While current recommendations for gunshot wound management involve conservative versus surgical management, we demonstrate a fortuitous situation involving successful and minimally invasive extraction followed by routine antibiotic and outpatient management.
